# Comprehensive Overview of Molecular, Imaging, and Therapeutic Challenges in Rectal Mucinous Adenocarcinoma

**DOI:** 10.3390/ijms26020432

**Published:** 2025-01-07

**Authors:** Mihaela Berar, Andra Ciocan, Emil Moiș, Luminița Furcea, Călin Popa, Răzvan Alexandru Ciocan, Florin Zaharie, Cosmin Puia, Nadim Al Hajjar, Cosmin Caraiani, Ioana Rusu, Florin Graur

**Affiliations:** 1 3rd Department of Surgery, “Iuliu Hațieganu” University of Medicine and Pharmacy, 400162 Cluj-Napoca, Romania; dragota.maria.mihaela@elearn.umfcluj.ro (M.B.); drmoisemil@elearn.umfcluj.ro (E.M.); luminita.furcea@umfcluj.ro (L.F.); cpopa@elearn.umfcluj.ro (C.P.); zaharie.vasile@umfcluj.ro (F.Z.); drpuia@yahoo.fr (C.P.); nadim.alhajjar@umfcluj.ro (N.A.H.); florin.graur@umfcluj.ro (F.G.); 2 Octavian Fodor Regional Institute of Gastroenterology and Hepatology, 400162 Cluj-Napoca, Romania; ioana.russu@yahoo.com; 3 Department of Surgery-Practical Abilities, “Iuliu Hațieganu” University of Medicine and Pharmacy, 400337 Cluj-Napoca, Romania; razvan.ciocan@umfcluj.ro; 4 Department of Medical Imaging, “Iuliu Hațieganu” University of Medicine and Pharmacy, 400006 Cluj-Napoca, Romania; ccaraiani@yahoo.com

**Keywords:** mucinous adenocarcinoma, rectal cancer, neoadjuvant treatment, molecular pathways

## Abstract

Rectal cancer is one of the most frequent malignancies worldwide. The most common histological type is adenocarcinoma, followed by mucinous adenocarcinoma. The outcome is less favorable for the mucinous type, yet the treatment course is the same. The aim of this systematic literature review is to assess existing information in order to improve survival in rectal mucinous adenocarcinoma (RMA) and establish a starting point for future research. A systematic search of PubMed, Google Scholar, and Web of Science online libraries was performed in October 2024, evaluating studies regarding clinicopathological and genetic features in connection with targeted treatment and survival outcomes in RMA, using the terms “rectal cancer”, “rectum”, “mucinous adenocarcinoma”, or a combination of the terms. We selected 23 studies, 10 of them regarding the diagnostic implications and 13 discussing the treatment strategies and prognosis of this histological subtype. There were six studies addressing the imaging aspects, highlighting the distinct features of mucinous histology in MRI. The molecular specifics were detailed in four studies, outlining the molecular footprint. The prognosis and treatment course were addressed in 12 studies. The inflammation index prognosis, complete response to neoadjuvant chemotherapy, and surgical aspects were addressed individually in each study. We encapsulated the molecular and clinicopathological characteristics of RMA, as well as diagnostic and treatment approaches, to establish a baseline of references for the benefit of daily practice and further research.

## 1. Introduction

Colorectal cancer ranks as one of the most prevalent malignancies globally, with the third highest incidence of all types of malignancy. Histologically, rectal cancer encompasses several subtypes, including adenocarcinoma, mucinous (colloid) adenocarcinoma, adenosquamous carcinoma, squamous cell carcinoma, and small-cell carcinoma. The predominant subtype is adenocarcinoma, originating from the epithelial cells of the mucosa, accounting for around 90% of cases. Rectal mucinous adenocarcinoma (RMA) is a rare but distinctive histological form of colorectal cancer. Although classified as a subtype of rectal adenocarcinoma, it has distinct features that subsequently influence its prognosis. It constitutes around 10–15% of all cases of rectal cancer. The histopathological characteristic of the mucinous subtype is the presence of extracellular mucin comprising over 50% of the tumoral tissue. This variant, characterized by elevated production of extracellular mucin, has unique challenges for diagnosis, treatment, and prognosis. Mucinous tumors are associated with more aggressive conduct, resistance to standard treatment, and worse clinical outcomes due to the natural evolution of the disease. This contrasts with the more prevalent forms of rectal adenocarcinoma, despite sharing similarities in clinical presentation. Consequently, to provide optimal therapy for patients, it is essential to possess a comprehensive understanding of the clinical features, molecular biology, and therapeutic strategies specific to rectal mucinous adenocarcinoma. Comprising 5–20% of all colonic and rectal malignancies, its prevalence varies by region: in Western nations like the US, it accounts for 11%, but in Japan, it is as low as 3% [1]. Studies indicate that women are at an elevated risk of developing mucinous adenocarcinoma (MAC) [2], and age is a significant determinant in certain groups. Huang et al. [1] showed that Chinese patients under 50 years of age are at a higher risk of developing MAC, while research conducted in the USA indicated an elevated risk for individuals over 65 years of age [2].

This article aims to provide an overview of fundamental characteristics, diagnostic criteria, and prognosis based on specific gene alterations, pelvic MRI diffusion imaging, and clinical response to oncological treatment according to the stage at the time of diagnosis, with a focus on facilitating clinical practice and offering a more efficient pathway for targeted treatment. Moreover, it underscores the need for innovative treatment strategies customized to the unique biological and radiological attributes of RMA, hence promoting progress in clinical care and translational research.

## 2. Methods

For this systematic literature review, we performed an electronic search using PubMed, Google Scholar, and the Web of Science databases. The investigation was conducted in October 2024, assessing articles published in the English language about the genetic and molecular characteristics, clinicopathological features, treatment modalities, and survival outcomes of rectal mucinous adenocarcinoma. The search used the phrases “mucinous adenocarcinoma” and “rectal cancer” or “tumor of the rectum” or a combination of them.

The chosen articles were required to be original articles published from 2015 until 2024, open access, and to include human subjects. Studies not published in English, review papers, meta-analyses, brief reports, or letters to the editor were omitted from our evaluation. The article seeks to provide a comprehensive examination of the original results and evidence obtained from primary research investigations. Excluding review and meta-analysis articles guarantees that the emphasis stays on primary data sources. The article focuses on original research, hence avoiding repetitive reporting. Limiting the inclusion criteria to primary research maintains the article’s focus and manageability, allowing a more thorough examination of pertinent results without straying into secondary literature; moreover, it facilitates a more direct and novel analysis of the primary data.

**Figure 1:**
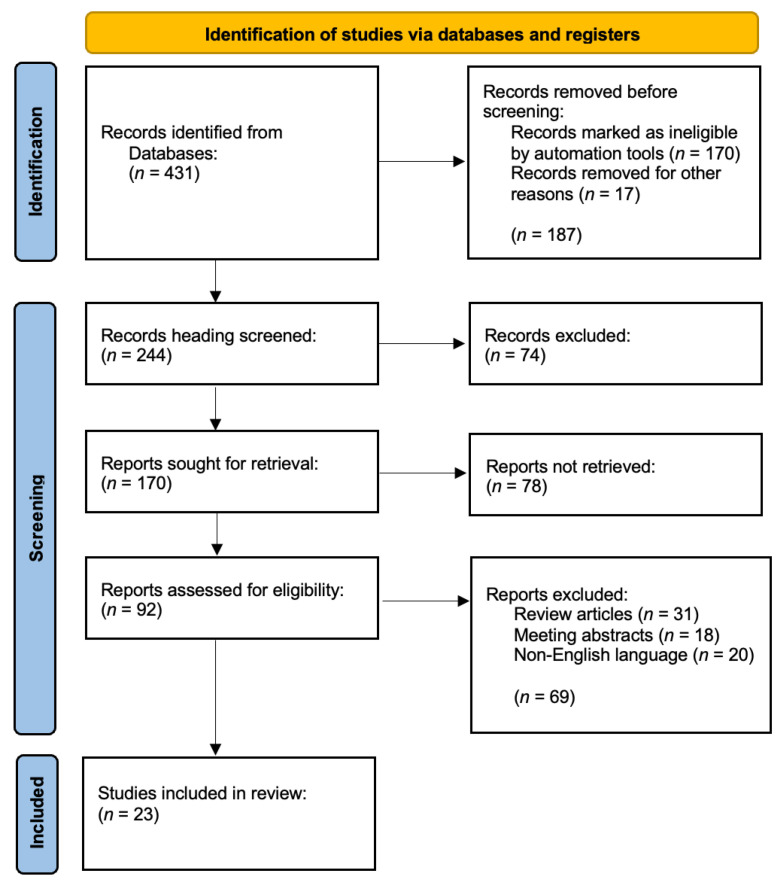
PRISMA flowchart of the included studies.

To conduct a thorough evaluation of bias risk in each study, we used the updated Quality Assessment of Diagnostic Accuracy Studies-2 (QUADAS-2) tool (accessed on 1 November 2024), which encompasses four bias domains: patient selection, index test, reference standard, and patient flow and timing. A Microsoft Excel extraction tool from Microsoft Office 2021 was used to organize and gather data. Two separate reviewers assessed all chosen articles, with final consensus judgements incorporating a third reviewer as required, to meticulously choose suitable publications for inclusion in this study (Figure 1). The QUADAS-2 tool and other quality evaluation frameworks are optimally used for primary diagnostic accuracy studies, with them being versatile and resilient instruments that enhance the assessment of diagnostic accuracy investigations, hence elevating the quality and dependability of diagnostic research.

A total of 431 results were collected, 187 of which were excluded prior to screening due to irrelevance to human disease, lack of relation to the rectum, or focus on other issues. Of the 244 records, 152 publications were eliminated owing to irrelevance, being case reports, treating other malignancies, or being published over 15 years ago. The review included 23 studies, having rejected 69 papers for reasons including non-English language (20 articles), review-type articles (31 articles), and conference abstracts (18 articles), to avoid redundancy and provide up-to-date findings.

**Figure 2:**
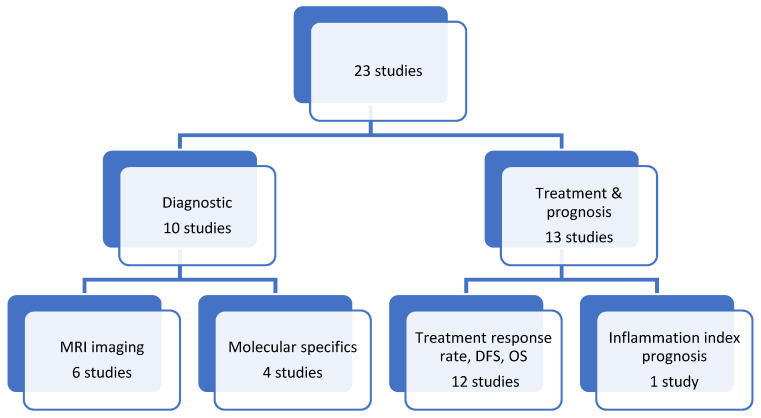
Subject flowchart of the studies included.

Out of the 23 studies included in this study, 10 publications addressed diagnostic difficulties. Four papers examined the molecular characteristics of the mucinous subtype, whereas six articles addressed the imaging features. We summarized thirteen papers concerning treatment modalities and prognosis, as shown below: one paper examined the prognosis of the inflammation index, while twelve papers analyzed the treatment response rate, disease-free survival, and overall survival, typically in comparison with rectal adenocarcinoma. One paper addressed the comprehensive response to neoadjuvant chemoradiotherapy, whereas another concentrated on the surgical dimensions of the disease, as seen in Figure 2.

## 3. Results and Discussion

### 3.1. Molecular Dimensions

An overview of the research concerning diagnostic features is shown in Table 1. Melis et al. [3] performed research using tissue microarray analysis to compare mucinous and non-mucinous adenocarcinomas and identified that MUC5AC was a significantly overexpressed gene in the mucinous samples compared to the non-mucinous samples. MUC2 and MUC5 were upregulated, and Aquaporins (AQP1, AQP3, and AQP5) were overexpressed, resulting in enhanced production of glandular fluid. This factor may disrupt metabolic turnover and metastatic potential, stimulating uncontrolled cellular proliferation and enhancing metastatic dissemination of MAC and resistance to treatment. Furthermore, the processes related to mucin formation, including O-glycan biosynthesis and keratan sulfate metabolism, were elevated.

Reynolds et al. [4] analyzed the molecular characteristics of MAC and non-MAC tumors. The mucinous cohort had an elevated likelihood of possessing altered genes associated with mucin glycoproteins, including MUC5B, MUC6, MUC14, and MUC18. Microsatellite instability is a contributing factor to the development of mucinous adenocarcinoma. Genes that are underexpressed, including MLH1, APC, and p53, are key factors in the pathogenesis of the disease.

Reynolds et al. [5] performed research including 10 patients with rectal mucinous adenocarcinoma and 10 patients with non-mucinous adenocarcinoma, whose complete genomes were sequenced. The final results indicated an elevated mutation rate of APC (80%), KRAS (70%), FBXW7 (50%), TP53 (40%), and MUC16 (40%) across all patients. However, when microsatellite stability (MSI) was analyzed, the mucinous MSI group exhibited a KRAS mutation rate of 69.2%, in contrast with the adenocarcinoma MSI group, who presented a statistically significant higher mutation rate of 35.3% (*p* = 0.03). During further comparison of the two cohorts, PIK2CA and MUC16 mutations were prevalent (30.8% vs. 8.8%, *p* < 0.05 and 30.8% vs. 0.0%, *p* < 0.001, respectively). Moreover, BRAF and KRAS, together with the presence of *Fusobacterium nucleatum*, may contribute to the suboptimal response to chemotherapy in these tumors by stimulating TNFα production. The bacterium is linked to tumors with an unfavorable prognosis owing to their MSI-H and MLH-1 status, in addition to their CpG Island Methylator status.

Lan et al. [6] examined the molecular characteristics of 20 MAC tumors among 125 rectal cancer patients and found no differences between MAC and non-MAC tumors.

### 3.2. Imaging Aspects

Six investigations elucidated the imaging characteristics of rectal mucinous carcinoma, as presented in Table 1. Yu et al. [7] emphasized the significance of MRI imaging in detecting the mucinous subtype, noting that MRI diagnostic rates surpassed those of biopsy, while Nasu et al. [8] demonstrated that various MRI protocols can differentiate mucinous tumors from tubular ones. Consequently, the elevated mucin content in MAC patients results in T2-weighted images exhibiting a high-intensity signal, similar to that of mesorectal fat. This may be regarded as a pathognomonic marker. The TW2I sequences may reveal a tumor encircled by a dark rim, resembling the internal sphincter complex (Adnexa A). Contrast-enhanced images may be used to distinguish MAC from non-MAC tumors, using T2-weighted imaging with fat suppression and diffusion-weighted imaging. Diffusion-weighted images depict this rectal cancer subtype as a low-intensity signal when compared to adenocarcinoma, due to the mucinous component resulting in reduced cellularity. Measuring the ADC (apparent diffusion coefficient) allows for differentiation between various rectal types of tumors and rectal MAC, with the latter exhibiting a greater ADC [8,9] (Supplementary Materials S2).

Turkish researchers [9] determined that the apparent diffusion coefficient (ADC) may distinguish mucinous carcinoma from adenocarcinoma due to their distinct diffusion properties. Miyakita et al. [10] demonstrated that the proportions of mucinous components seen on MRI at the moment of diagnosis serve as a predictor of response. Enlarged mucin pools correlate with increased tumoral growth and higher resistance to neoadjuvant therapy [10]. Koëter et al. [11] highlighted the inadequate response of MAC to neoadjuvant therapy from an imaging perspective. Queiroz et al. [12] assessed tumor metabolism in their research contrasting MAC with non-MAC PET-MRI features, indicating a reduction in glycolytic metabolism within the mucinous group. These tumors exhibit reduced fluorodeoxyglucose absorption owing to their diminished cellularity and resultant poorer glucose metabolism.

### 3.3. Management and Outlook

Twelve publications examined treatment choices and prognosis, whereas one study investigated inflammatory scores as potential predictors of treatment response, as seen in Table 2. All studies compared the mucinous subtype to adenocarcinoma in terms of overall survival, disease-free survival, and tumor response to neoadjuvant or adjuvant therapy regimes. The current approach of neoadjuvant treatment for individuals diagnosed with rectal MAC has a suboptimal response rate.

The mucinous subtype had fewer favorable results [13,14,15,16,17,18,19,20,21,22,23]. Simha et al. [14] determined the residual tumor following preoperative chemoradiotherapy as T4 in 73.5% of rectal mucinous adenocarcinoma cases, in contrast to 10.1% in the non-mucinous cohort. Additionally, the incidence of residual lymph node invasion was 29.4% compared to 9.3% in the non-mucinous group, and the rate of pathological complete response was 0%, whereas the non-MAC group exhibited a complete response rate of 11.7%. Hosseini et al. [19] identified that mucinous histology serves as a predictive factor for a suboptimal response to neoadjuvant chemotherapy by utilizing Capecitabine. Furthermore, Li et al. [20] determined that only patients with stage II rectal mucinous adenocarcinoma will gain benefit from postoperative (adjuvant) radio-chemotherapy. Also, patients undergoing a long-course radiation regimen with mucinous histology presented no progression, in contrast with the non-mucinous cohort, who developed a 7% progression rate.

The surgical considerations examined in an article by Huang et al. [21] indicated that the laparoscopic technique is a viable choice for the mucinous subtype, while the inflammatory scores suggested by Sun et al. [25] may predict the response to radio-chemotherapy.

Disease-free survival is affected by tumor downstaging rate after neoadjuvant treatment, adjuvant therapy, and a lower clinical stage, while overall survival is associated with the histological subtype, pathological full response, and pathologic tumor stage in Awad et al.’s study [22]. Statistical differences were observed in stages IIIa and IIIb between the mucinous group receiving adjuvant chemotherapy, first regimens (FOLFOX/CAPEOX/FOLFIRINOX and radiation), and those not receiving it, in favor of the chemotherapy group. In the event of tumor progression or lack of therapeutic response, additional immunotherapy is necessary (bevacizumab/cetuximab/panitumumab).

Sun et al. [25] considered that reduced pre-treatment levels of the systemic immune-inflammation index, platelet-to-lymphocyte ratio, and neutrophil-to-lymphocyte ratio relate to an increased likelihood of a favorable response to treatment in MAC.

It is documented that the development of MAC usually develops from serrated polyps; nevertheless, Melis et al. [3] identified newly upregulated pathways that may suggest that MAC has a distinct molecular signature. The tertiary structures of proteins are influenced by the overexpression of sialyltransferase, thereby diminishing the immunological response. The mucin-producing molecules fail to complete their glycosylation process, enabling malignant cells to evade the organism’s defensive mechanisms by inhibiting T-cell synthesis [3].

MSI plays a key in mucinous adenocarcinoma, with these patients exhibiting a higher KRAS mutation rate compared to adenocarcinoma patients, similar to PIK2CA and MUC16. Some instances had shared genetic and molecular routes with colon cancer, whereas others resembled skin cancer, lymphoma, and leukemia. Moreover, the failure of DNA double-strand repair linked to BRCA1 and BRCA2 mutations is rare but with a specific molecular route [5]. Nonetheless, the differential expression of MLH1, MSH2, and MLH6, although resulting in excessive mucin synthesis, does not always correlate with local recurrence [26]. This underscores the polymorphic characteristics of the mucinous subtype (Supplementary Materials S1) and the necessity for a tailored approach to neoadjuvant or adjuvant systemic therapy, given the established lack of response to chemotherapies involving 5-fluorouracil) in cases exhibiting diminished thymidine phosphorylase expression [27].

Furthermore, the integration of genomic and microbiological analyses seems to enhance the comprehension of molecular processes. Certain bacterial species, including *Fusobacterium nucleatum*, have been associated with rectal tumor microenvironments exhibiting a poor response to treatment. The existence of *F. nucleatum* seems to be associated with mucin formation since the bacterium stimulates MUC2 and TNFα production. The mechanism of medication resistance seems to entail modulated autophagy. Certain experimental studies have shown that simply eliminating the bacteria from the tumoral microbiota by administering Metronidazole in mice results in an important reduction in neoplastic growth rates [28].

The gold standard method for staging rectal cancer is pelvic MRI, which may also serve as a diagnostic instrument. Delineating the tumor is crucial for precise cancer staging [29]. An ongoing controversy exists about the reliability of MRI in ascertaining the histology of MAC. Kim et al. [30] asserted that MRI specificity in distinguishing mucinous adenocarcinoma from regular adenocarcinoma reaches 97%, and Yu et al. [7] endorse the notion that MRI excels in identifying this histology preoperatively in contrast with the established protocol of histological confirmation of the mucinous adenocarcinoma subtype. Proponents of the imagistic technique argue that biopsies are often obtained from the surface region of the tumor, resulting in insufficient cellularity retrieval and subsequent histological misdiagnosis [8] (Supplementary Materials S1).

In addition to its diagnostic capabilities, MRI imaging in MAC may also serve as a prognostic indicator. Nevertheless, the increased mucin characteristic may be deceptive. Multiple factors contribute to false positive findings, including necrosis, edema, congestion, and necrosis, which may not be distinguishable from this viewpoint [29]. Following neoadjuvant treatment, acellular mucin, including aggregates of mucin devoid of viable tumor cells, indicates a good response to treatment [31]. Distinguishing between cellular and acellular mucin with MRI is rather challenging. Despite the absence of acellular mucin affecting patients’ disease-free survival or overall survival [11,32], it is crucial to conduct diligent follow-up on these patients, since mucin pools may be infiltrative, hence lowering the likelihood of achieving an R0 surgical resection [31].

Miranda et al. analyzed the progress and effectiveness of MRI in assessing rectal cancer post-neoadjuvant treatment. The results emphasize the essential function of MRI in treatment planning and outcome forecasting, showcasing its enhanced precision and detail relative to other imaging techniques.

Other imaging modalities, such as contrast-enhanced CT scans, are mostly used for assessing distant metastasis rather than local invasion. Endorectal ultrasonography has comparable accuracy to pelvic MRI in local staging and may detect lesions overlooked by MRI, particularly in early stages (T1), or in the post-neoadjuvant context, when differentiating residual malignant tissue from fibrosis proves challenging [33]. Other investigations have shown that there is no substantial difference between mucinous and non-mucinous in regard to PET-MRI characteristics [34]. Radiomics is an advanced technique that involves extracting and analyzing a wide range of quantitative features from medical images, such as size, shape, texture, intensity, and spatial relationships within the image structures [35]. A meta-analysis by Tanaka et al. [26], who reviewed 16 studies, found that radiomic models show promising potential for predicting the response to neoadjuvant therapy in rectal cancer patients. Integrating molecular imaging biomarkers corresponds with the increasing focus on individualized cancer treatment. They enable the selection of suitable therapy modalities, such as choosing between surgical and non-surgical alternatives like the “watch-and-wait” strategy, informed by predictive imaging data [36].

Ultimately, despite rectal MAC being a distinct entity with unique oncological pathways and a more aggressive progression, the therapeutic choices remain the same with the regimens for adenocarcinoma. Although the National Comprehensive Cancer Network (NCCN) does not recommend different therapeutical algorithms, several studies have shown a distinct patient profile associated with poor outcomes [17,37]. At the time of diagnosis, rectal MAC often presents with a greater tumor burden and is often identified at an advanced stage, generally stage III [38]. Mucinous rectal histology serves as an independent unfavorable prognostic factor for stage II cancer in individuals under 55 years of age [17].

The established treatment protocol for locally advanced rectal cancer includes comprehensive neoadjuvant chemoradiotherapy, surgical intervention, and adjuvant treatment. The pathological complete response was diminished in the mucinous adenocarcinoma cohort compared to other rectal cancer histological subtypes [39]. Moreover, Hosseini et al. [18] concluded that stage II cancer seems to have a superior response rate to neoadjuvant treatment in contrast with more advanced stages. Notwithstanding the persistent discourse, the radiation protocol in place at this point remains unchanged [40].

Sun et al. [25] showed that an elevated prognostic nutritional index, which integrates lymphocyte count and albumin levels, correlates with a favorable result after neoadjuvant therapy [41].

The surgical strategy for all rectal cancer patients entails full resection with negative specimen margins and complete mesorectal excision [42]. Regarding concerns raised about the laparoscopic approach regarding the infiltrative spread of mucin, which possesses a higher risk of peritoneal contamination, Huang et al. [24] proved its feasibility, by compensating its increased operative time with reduced postoperative complications, and shorter patient recovery time, enabling the patients to start adjuvant therapy faster. Furthermore, robotic surgery for colorectal cancer has become more accessible [43,44]. Consequently, in complicated tumors producing bowel obstruction, perforation, and hemorrhage, open surgery should still be considered as the gold standard.

Adjuvant treatment comprises 5-fluorouracil-based regimens, but certain studies assert that only individuals with stage II cancer gain benefit from it, increasing disease-free survival but not overall survival [19,45]. The FOLFOX and CAPEOX procedures were developed to enhance overall survival, with little to no difference in treatment response being exhibited between the two protocols. Certain authors assert that rectal mucinous histology does not influence stage I disease, having no effect on patient prognosis or tumor recurrence compared to stage III of the disease, advocating for a more tailored approach [13], noted also by Kim et al. [22]. A Swedish study [46] found no differences in overall survival and disease-free survival between mucinous and non-mucinous rectal cancer.

Immunotherapy was administered to individuals with advanced, metastatic colorectal mucinous adenocarcinoma with microsatellite instability (MSI) or deficient mismatch repair (dMMR). The focus was on the anti-epidermal growth factor—Cetuximab—and vascular endothelial growth factor (VEGF)—Bevacizumab. NCCN recommendations endorse Pembrolizumab as the primary treatment for unresectable or metastatic cancer if intense chemotherapy is not advised [27,28,29,40].

The mucinous subtype poses a significant risk of intraperitoneal spread, prompting the use of hyperthermic intraperitoneal chemotherapy (HIPEC) to eradicate microscopic tumor remains, without any consent on drug exposure duration and medicine dilution [47]. It is essential to evaluate the risks associated with the surgery, since intestinal perforation, electrolyte imbalance, and renal failure are common occurrences [48].

However, the review presents its limitations related to secondary research. Numerous investigations concerning rectal mucinous adenocarcinoma are retrospective observational studies, susceptible to selection bias and potentially lacking control for confounding variables. Secondly, research often encompasses varied populations, integrating MAC with non-mucinous subtypes or amalgamating early-stage and advanced-stage patients. This diversity may obscure insights pertinent to the distinct biology and behavior of MAC. There exists considerable variety in the imaging modalities used between studies (MRI vs. PET), and the absence of standardized imaging methods may result in discrepancies in histological tumor characterization. Differences in the definition of mucinous adenocarcinoma (e.g., minimum mucin content threshold) lead to inconsistencies in patient categorization and research results.

## 4. Conclusions

Raising concern about the specifics of the molecular pathway in rectal mucinous adenocarcinoma, despite its similarities to rectal adenocarcinoma, poses pivotal clinical importance in regards to the distinct response to surgical and systemic treatment with overall lower survival and poorer prognosis. MAC derives from serrated polyps overexpressing MUC2, MUC5, and Aquaporins. Mucin overproduction evades T-cell immune response, resulting in uncontrolled cell proliferation and metastatic dissemination. *Fusobacterium nucleatum*’s presence in the tumoral microenvironment further stimulates MUC2 and TNFα production with decreased response to neoadjuvant treatment in stages II and III of the disease.

T2-weighted imaging with fat suppression and diffusion-weighted imaging on pelvic MRI are useful tools in distinguishing mucinous from non-mucinous types of rectal cancer. ADC measurement significantly increases specificity demonstrated on biopsy, which can aid clinicians in the early selection of accurate oncological treatment options in stages II and III of the disease when the response is significantly lower than in other subtypes of adenocarcinoma. Future research should concentrate on the integration of sophisticated MRI methodologies with novel therapeutic strategies to enhance patient outcomes. Immunotherapy is often used as a last resort for cancer patients, but targeted therapy, aimed at particular overexpressed protooncogenes, seems to point toward the future direction for drug development.

## Figures and Tables

**Table 1:** Molecular and imaging characteristics of mucinous rectal cancer.

Author	Year	Total Patients	MAC Patients	Major Findings
Molecular aspects				
Melis et al. [3]	2019	171	20	Altered biologic pathways included those associated with the metabolism of mucin amino-acid and amino acids and the mitogen-activated protein kinase cascade. The most upregulated genes are those participating in cellular differentiation and mucin metabolism.
Reynolds et al. [4]	2019	530	67	The genetic alterations in mucinous tumors are determined by microsatellite instability (MSI-H) and also affect the genes responsible for the mucin glycoproteins.
Reynolds et al. [5]	2020	10	10	Mucinous tumors that have BRAF, KRAS, and MUC16 mutations are connected with the persistence of anaerobic bacteria in the tumoral microenvironment, leading to chemotherapy resistance.
Lan et al. [6]	2021	125	20	No significant difference in genetic mutations when comparing rectal MAC and non-MAC.
Imaging aspects				
Yu et al. [7]	2014	330	60	Initial MRI evaluation of rectal mucinous tumors was more accurate in diagnosing the mucinous subtype raising concern before biopsy and could therefore guide the treatment scheme.
Nasu et al. [8]	2012	81	15	Rectal MAC can be differentiated from tubular adenocarcinoma using different MRI protocols.
Er et al. [9]	2017	62	18	Different histological subtypes, such as rectal MAC, can be individualized by their diffusion characteristics.
Miyakita et al. [10]	2018	205	20	Mucin pool percentage described on MRI reflects the neoadjuvant treatment response.
Koeter et al. [11]	2021	102	29	MRI characteristics of rectal mucinous tumors after neoadjuvant radio-chemotherapy can be used as prognostic markers.
Queiroz et al. [12]	2020	99	17	PET MRI can be a useful tool in evaluating the glycolic metabolism in mucinous rectal tumors.

MAC—mucinous adenocarcinoma; MSI-H—high-grade microsatellite instability or mismatch repair deficient (dMMR); BRAF—serine/threonine-protein kinase (protooncogene); KRAS—Kirsten rat sarcoma virus (protooncogene); MUC16—part of the mucin family glycoproteins (CA125—ovarian cancer associated tumoral marker); MRI—magnetic resonance imaging; PET—positron emission tomography.

**Table 2:** Survival and recurrence risk in MAC.

Author	Year	Total Patients	MAC Patients	Major Findings
Bong et al. [13]	2022	7690	196	No major survival differences between adenocarcinoma and mucinous adenocarcinoma.
Simha et al. [14]	2014	162	34	Treatment response was poorer for mucinous patients compared to those with the non-mucinous type.
Numata et al. [15]	2021	1214	57	Stage III disease patients with mucinous histology are more likely to have local recurrence and peritoneal metastases than non-MAC patients.
Grillo-Ruggieri et al. [16]	2007	136	25	Mucinous histology is associated with a lower downstaging rate after neoadjuvant treatment.
Venmark et al. [17]	2020	433	54	Overall survival was poor for patients with the mucinous subtype. However, patients with long-course radiotherapy presenting tumor regression had better overall survival than patients with no regression.
Wuet et al. [18]	2020	10910	633	Young patients (<55 years) with stage II mucinous histology have poorer prognosis compared to non-MAC patients.
Hosseini et al. [19]	2019	403	46	Mucinous histology is an independent factor for low-rate pathological complete response in patients with locally advanced rectal cancer who undergo capecitabine-based chemotherapy.
Li et al. [20]	2017	574	574	Adjuvant radiotherapy is an independent favorable prognostic factor for patients with stage II disease, but for stage III patients, caution should be taken due to the increased number of side effects and no case-specific survival benefit.
Huang et al. [21]	2022	530	58	Histology is not an independent predictor factor for recurrence or prognosis in stage I disease.
Awad et al. [22]	2021	70	17	Mucinous histology is a poor prognostic factor for overall survival and disease-free survival in stage II and III disease.
Kim et al. [23]	2016	412	30	Rectal mucinous histology is associated with a poorer prognosis and worse response rate compared to rectal adenocarcinoma.
Huang et al. [24]	2021	162	48	The laparoscopic approach is a feasible option for surgical resection in MAC.
Sun et al. [25]	2020	128	128	Immunonutritional indexes such as albumin-to-globulin ratio and prognostic nutritional index could predict treatment response.

MAC—mucinous adenocarcinoma; stages I, II, and III of rectal cancer based on the TNM classification of the AJCC 8th Edition.

## Data Availability

The original contributions presented in this study are included in the article/Supplementary Material [49].

## Supplementary Materials

The following supporting information can be downloaded at: https://www.mdpi.com/article/10.3390/ijms26020432/s1.
