# Supplementary material for: Comprehensive Overview of Molecular, Imaging, and Therapeutic Challenges in Rectal Mucinous Adenocarcinoma
Source: Int J Mol Sci. 2025 Jan 7;26(2):432. doi: 10.3390/ijms26020432 (PMC11764815; doi:10.3390/ijms26020432)
Supplement: Supplementary file 1 [file ijms-26-00432-s001.zip › ijms-3352885-Supplementary materials S1.pdf]

International Journal of *Molecular Sciences*

Review

Comprehensive Overview of Molecular, Imaging, and Therapeutic Challenges in Rectal Mucinous Adenocarcinoma

Mihaela Berar, Andra Ciocan, Emil Moiș, Luminița Furcea, Călin Popa, Răzvan Alexandru Ciocan, Florin Zaharie, Cosmin Ion Puia, Nadim Al Hajjar, Cosmin Caraiani, Ioana Rusu and Florin Graur

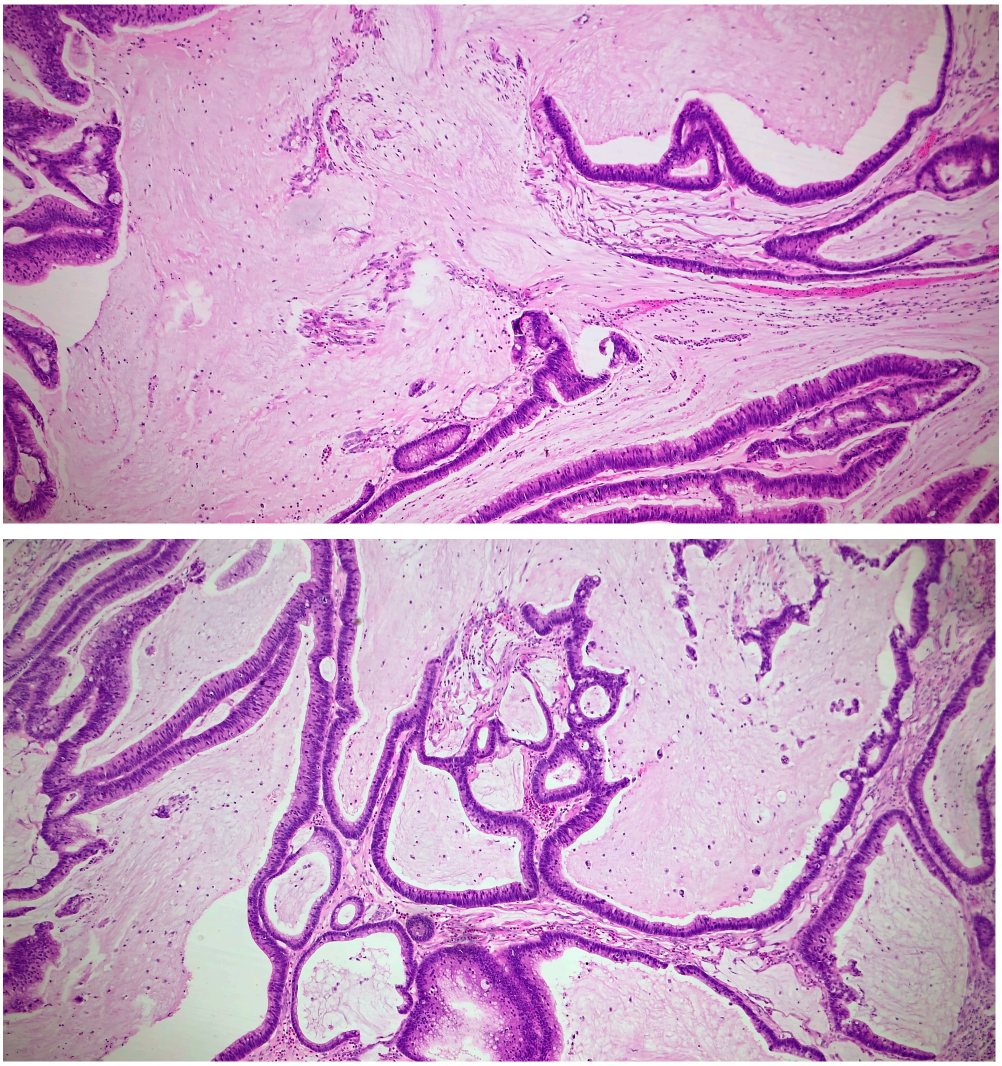

Figure S1.1. Mucinous adenocarcinoma in hematoxylin-eosin 10X magnification – showing large mucin pools

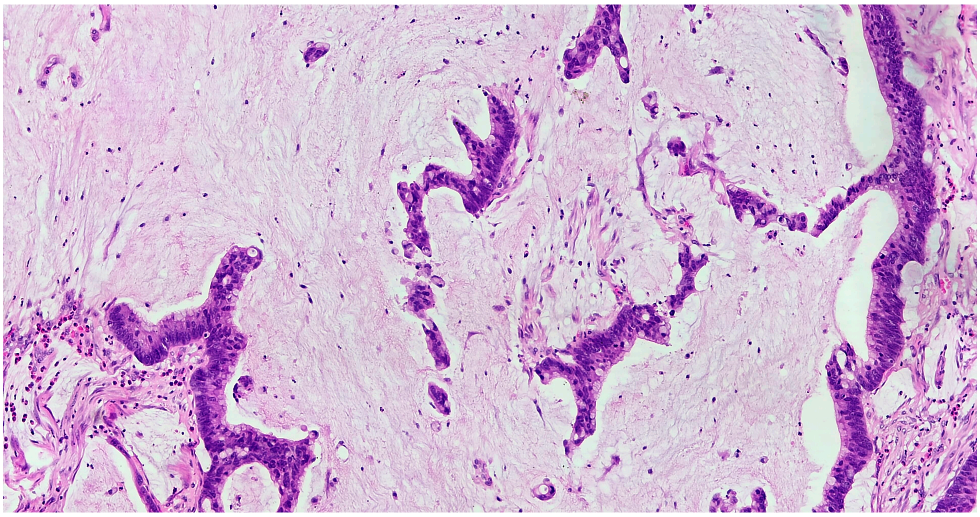

Figure S1.2. Mucinous adenocarcinoma–hematoxylin-eosin stained biopsy 10X magnification showing abundant extracellular mucin
