# Supplementary material for: Comprehensive Overview of Molecular, Imaging, and Therapeutic Challenges in Rectal Mucinous Adenocarcinoma
Source: Int J Mol Sci. 2025 Jan 7;26(2):432. doi: 10.3390/ijms26020432 (PMC11764815; doi:10.3390/ijms26020432)
Supplement: Supplementary file 1 [file ijms-26-00432-s001.zip › ijms-3352885-Supplementary materials S2.pdf]

International Journal of
Molecular Sciences

MDPI

Review

Comprehensive Overview of Molecular, Imaging, and Therapeutic Challenges in Rectal Mucinous Adenocarcinoma

Mihaela Berar, Andra Ciocan, Emil Moiș, Luminița Furcea, Călin Popa, Răzvan Alexandru Ciocan, Florin Zaharie, Cosmin Ion Puia, Nadim Al Hajjar, Cosmin Caraiani, Ioana Rusu and Florin Graur

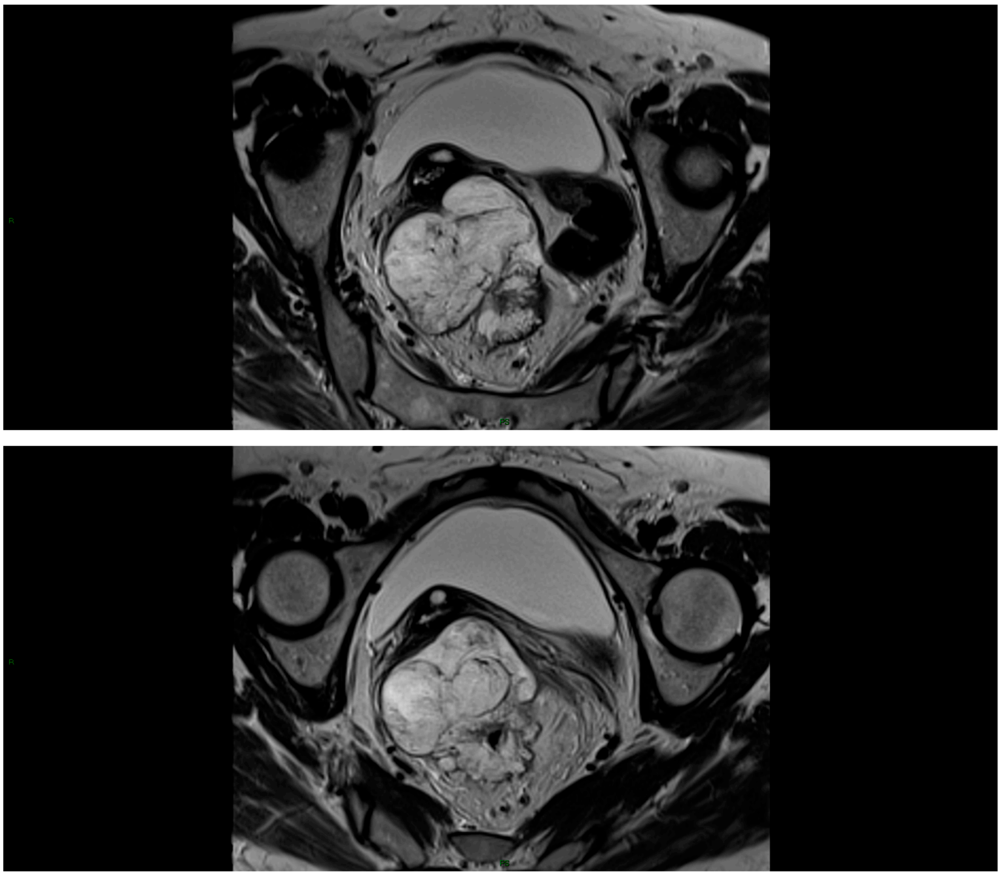

Figure S2.1. T2-weighted MRI images of mucinous adenocarcinoma – axial section showing mesorectal invasion of the tumor

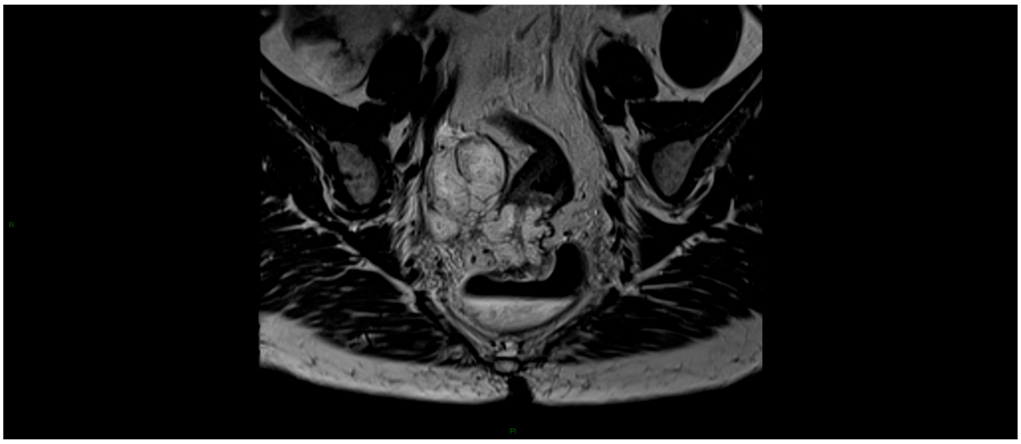

Figure S2.2. T2-weighted MRI images of mucinous adenocarcinoma – coronal section showing locally T2-hyperintense signal greater than 50%

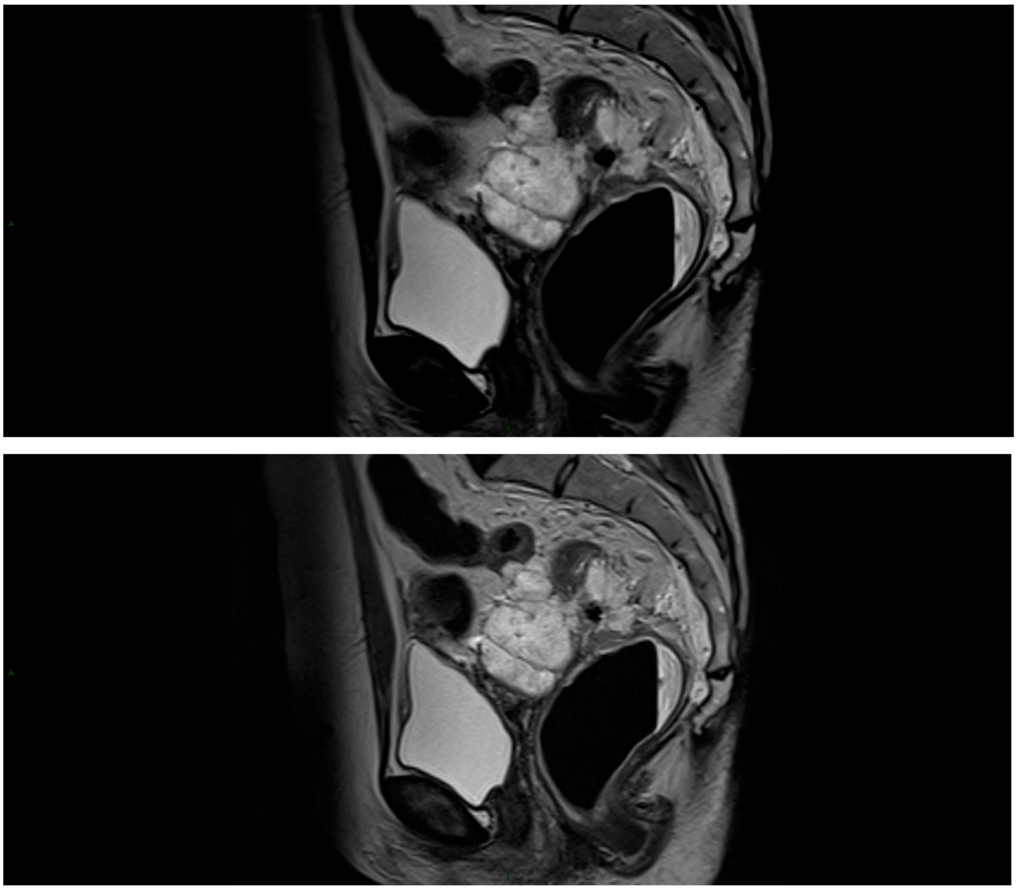

Figure S2.3. T2-weighted MRI images of mucinous adenocarcinoma – sagittal section showing locally advanced cancer, ADC map revealed diffusion restriction
